# PET Imaging in Recurrent Medullary Thyroid Carcinoma

**DOI:** 10.1155/2012/324686

**Published:** 2012-07-18

**Authors:** Giorgio Treglia, Vittoria Rufini, Massimo Salvatori, Alessandro Giordano, Luca Giovanella

**Affiliations:** ^1^Institute of Nuclear Medicine, Catholic University of the Sacred Heart, 00168 Rome, Italy; ^2^Department of Nuclear Medicine and PET-CT Centre, Oncology Institute of Southern Switzerland, Street Ospedale 12, 6500 Bellinzona, Switzerland

## Abstract

*Purpose*. To perform an overview about the role of positron emission tomography (PET) or PET/computed tomography (PET/CT) using different radiopharmaceuticals in recurrent medullary thyroid carcinoma (MTC) based on biochemical findings (increased tumor marker levels after primary surgery). *Methods*. A comprehensive literature search of studies published in PubMed/MEDLINE, Scopus, and Embase databases through February 2012 regarding PET or PET/CT in patients with recurrent MTC was performed. *Results*. Twenty-nine studies comprising 714 patients with suspected recurrent MTC were retrieved. Twenty-seven articles evaluated the role of fluorine-18-fluorodeoxyglucose (FDG) PET or PET/CT in recurrent MTC with conflicting results. Diagnostic accuracy of FDG-PET and PET/CT increased in MTC patients with higher calcitonin and carcinoembryonic antigen values, suggesting that these imaging methods could be very useful in patients with more advanced and aggressive disease. Eight articles evaluated the role of fluorine-18-dihydroxyphenylalanine (FDOPA) PET or PET/CT in recurrent MTC reporting promising results. Overall, FDOPA seems to be superior but complementary compared to FDG in detecting recurrent MTC. Few studies evaluating other PET tracers are also discussed. *Conclusions*. PET radiopharmaceuticals reflect different metabolic pathways in MTC. FDOPA seems to be the most useful PET tracer in detecting recurrent MTC based on rising levels of tumor markers. FDG may complement FDOPA in patients with more aggressive MTC.

## 1. Introduction

Medullary thyroid carcinoma (MTC) is a slow-growing neuroendocrine tumor originating from parafollicular C cells. MTC accounts for approximately 5% of thyroid carcinomas, occurring in either sporadic (75% of cases) or familial forms (25% of cases). This tumor is frequently aggressive; most frequent sites of metastatic disease are cervical and mediastinal lymph nodes, lungs, liver, and bone. The main treatment for MTC is surgical resection that is the only strategy for potential cure; in patients with metastatic disease therapeutic options are limited as this tumor does not concentrate radioiodine and shows poor response to chemotherapy and radiation therapy [1]. Also targeted therapy with vandetanib seems to show promising results in the treatment of patients with metastatic/recurrent MTC [1].

Serum calcitonin represents the most sensitive and accurate tumor marker in the postoperative management and surveillance of MTC. In about one third of patients with MTC lesions also carcinoembryonic antigen (CEA) levels may be increased and this finding has prognostic significance, as increased CEA levels are characteristic of advanced forms when the tumor tends to dedifferentiation. Serum calcitonin and CEA doubling times are efficient tools for assessing tumor progression and are useful prognostic factors of survival in patients with MTC [1].

The early detection of recurrence represents an important step in the management of patients with MTC, because identifying recurrent tumor tissue impacts in patient outcome [1–4]. Conventional imaging modalities are often negative or inconclusive in presence of rising levels of tumor markers. Therefore, functional imaging with PET using different radiopharmaceuticals was explored as a way to detect MTC recurrence.

Fluorine-18-Fluorodeoxyglucose (FDG), a glucose analog, accumulates in neoplastic cells allowing scintigraphic visualization of tumors that use glucose as an energy source. FDG uptake in neoplastic cells correlates with poor differentiation and high proliferative activity. Neuroendocrine tumors usually show an indolent course, and consequently low FDG uptake [3, 4]. These tumors, however, when undergoing dedifferentiation become more aggressive and may show increased FDG uptake, and this is also the case in MTC as demonstrated by the immunoreactivity for KI-67 expression (KI-67 is a nuclear protein that is associated with cellular proliferation) in surgically removed lesions [3, 4].

Dihydroxyphenylalanine (DOPA) is an amino acid that is converted to dopamine by aromatic amino acid decarboxylase (AADC). Fluorine-18-DOPA (FDOPA) is taken up through ubiquitous transmembrane amino acid transporter systems that are significantly upregulated in neuroendocrine tumors, including MTC. This upregulation is presumably secondary to the increased activity of metabolic pathways involving the enzyme AADC which is a specific property of neuroendocrine tumors.

The aim of this paper is to perform an overview of the literature about the role of PET and PET/CT using different radiopharmaceuticals in patients with recurrent MTC based on biochemical findings (increased tumor marker levels after primary surgery).

## 2. Search Strategy and Data Abstraction

A comprehensive computer literature search of the PubMed/MEDLINE, Scopus and Embase databases was carried out to find relevant published articles on the role of PET or PET/CT using different radiopharmaceuticals in patients with recurrent MTC. We used a search algorithm based on a combination of the terms: (a) “PET” or “positron emission tomography” and (b) “medullary” or “thyroid”. No beginning date limit was used; the search was updated until February 29th 2012. To expand our search, references of the retrieved articles were also screened for additional studies. No language restriction was used.

Only those studies or subsets in studies that satisfied all of the following criteria were included: (a) PET or PET/CT performed in patients with suspected recurrent MTC after primary surgery; (b) sample size of at least 6 patients with MTC. The exclusion criteria were (a) articles not within the field of interest of this paper; (b) review articles, editorials or letters, comments, conference proceedings; (c) case reports or small case series (sample size of less than 6 patients with recurrent/residual MTC); (d) possible data overlap (in such cases the most complete article was included).

For each included study, information was collected concerning basic study (author names, journal, year of publication, and country of origin), patient characteristics (number of patients with suspected recurrent MTC performing PET or PET/CT, mean age, and sex), technical aspects (study design, device used, radiopharmaceutical used, injected dose, time interval between radiopharmaceutical injection and image acquisition, acquisition protocol, image analysis, and reference standard used), and diagnostic performance data (sensitivity and specificity). Patients evaluated with PET or PET/CT before primary surgery were excluded from the analysis. Only patients with a postoperative PET imaging were included.

## 3. Literature Data

Twenty-nine articles comprising 714 patients with suspected recurrent MTC were retrieved using the above cited criteria [5–33]. The characteristics of the included studies are presented in Table 1.

**(A) PET and PET/CT Using Fluorine-18-Fluorodeoxyglucose**

Twenty-seven articles evaluating the role of FDG-PET or PET/CT in patients with recurrent MTC were selected and retrieved from the literature (Tables 1 and 2) [5–8, 10, 12–33]. Other six articles were not included for possible data overlap [34–39]. Overall, the studies using FDG-PET or PET/CT have reported conflicting results about the diagnostic performance of these functional imaging methods in patients with suspected recurrent MTC. In particular, sensitivity of these methods ranged from 17% to 95% whereas specificity, when reported, ranged from 68% to 100% (Table 2). A possible explanation for these heterogeneous findings could be related to diversity between the studies in technical aspects (Table 2) and inclusion criteria (patients with known lesions versus patients with occult disease at conventional imaging methods; patients with slowly progressive disease versus patients with more aggressive disease) [40].

False negative results of FDG-PET and PET/CT could be related to small lesions or to the slow growth of neuroendocrine tumors. Both factors impact the diagnostic accuracy of these imaging modalities. False positive results also occurred by using FDG-PET and PET/CT, and were typically due to inflammatory lesions [3, 4, 40].

It should be noted that a significant number of recurrent MTC, based on rising levels of tumor markers, remained unidentified using FDG-PET or PET/CT. On the other hand, it should be considered that FDG-PET and PET/CT were often performed in patients with suspected recurrent MTC after negative conventional imaging studies, affecting the surgical management of patients with recurrent MTC when hypermetabolic lesions were detected [2–4, 40].

Based on literature findings, the diagnostic performance of FDG-PET or PET/CT in patients with recurrent MTC improved in patients with higher serum calcitonin and CEA levels [40]. Also, sensitivity of FDG-PET and PET/CT improved in patients with shorter tumor markers (calcitonin and CEA) doubling times [6, 10, 14, 16, 18], confirming the usefulness of these imaging methods in patients with more aggressive disease (with high glucose consumption and high FDG uptake) compared to those with slowly progressive disease (with low glucose consumption and low FDG uptake) [40].

FDG-PET or PET/CT were usually performed in the included studies if no disease sites were identified on conventional imaging in patients with biochemical evidence of MTC recurrence or if calcitonin levels were elevated out of proportion to minor disease found on conventional imaging. The diagnostic performance of FDG-PET and PET/CT in recurrent MTC increased whether patients with known lesions at conventional imaging were included in the study population, because functional abnormalities are usually detectable by FDG-PET or PET/CT when anatomical changes are already evident.

**(B) PET and PET/CT Using Fluorine-18-Dihydroxyphenylalanine**

Eight articles evaluating the role of FDOPA-PET or PET/CT in patients with recurrent MTC were selected and retrieved from the literature (Tables 1 and 3) [5, 6, 11, 13, 16, 18, 23, 30]. Another article was not included for possible data overlap [41]. Overall, the studies using FDOPA-PET or PET/CT have reported promising results in recurrent MTC. In particular sensitivity of these methods ranged from 47% to 83% (Table 3); however, FDOPA-PET or PET/CT modified the surgical management of a significant number of patients with recurrent MTC when positive, because these functional imaging methods were often performed in patients with suspected recurrent MTC based on rising tumor markers after negative conventional imaging studies.

Differences in technical aspects (Table 3) and inclusion criteria could explain the heterogeneity between studies about the sensitivity values reported. False positive results of FDOPA-PET or PET/CT in recurrent MTC are uncommon. On the other hand, possible causes of false negative results of FDOPA-PET or PET/CT should be kept in mind; they could be probably related to small MTC lesions or to dedifferentiation, both factors affecting the diagnostic accuracy of these imaging methods.

Based on literature findings, the diagnostic performance of FDOPA-PET or PET/CT in recurrent MTC improved in patients with higher serum calcitonin levels [5, 6, 11, 13, 16, 18, 23, 30].

Comparative analyses between FDOPA and FDG have shown better results with FDOPA in terms of sensitivity and specificity and a complementary role of the two radiopharmaceuticals in the assessment of recurrent MTC. The different behavior of FDOPA and FDG in recurrent MTC can be explained by their different uptake mechanisms that, in turn, reflect the different metabolic pathways of neuroendocrine cells, including MTC cells. FDOPA is a marker of amino acid decarboxylation that is a feature of the neuroendocrine origin of MTC; so, it can be assumed that a higher FDOPA uptake is related to a higher degree of cell differentiation, whereas a higher FDG uptake is related to a high proliferative activity and a poor differentiation.

In the study of Hoegerle et al. [30], 10 MTC patients underwent both FDOPA-PET and FDG-PET after thyroidectomy. The sensitivity of both methods on a per-patient-based analysis was the same (60%), with discordant results in two patients (discordance rate was 20%: one case was positive at FDOPA-PET and negative at FDG-PET, another case was positive at FDG-PET and negative at FDOPA-PET). Nevertheless, FDOPA-PET revealed more lymph nodal metastases on a per lesion-based analysis compared to FDG-PET [30].

In the study of Beuthien-Baumann et al. [23], 15 MTC patients underwent both FDOPA-PET and FDG-PET after thyroidectomy. The sensitivity of both methods on a per-patient-based analysis was the same (47%), with discordant results in most of the patients on a per lesion-based analysis [23].

Koopmans et al. [18] performed both PET methods in 17 patients with recurrent MTC, reporting a higher sensitivity of FDOPA-PET compared to FDG-PET on a per-patient-based analysis (62% versus 24%, resp.); furthermore, these authors found discordant results in 7/17 (41%) patients. In particular in 6 patients FDOPA-PET was positive and FDG-PET was negative for MTC recurrence [18].

In 2009 Beheshti et al. [16] found a superiority of FDOPA-PET/CT compared to FDG-PET/CT in 19 MTC patients evaluated after primary surgery (sensitivity on a per-patient-based analysis was 81% versus 58%, resp.). Discordant results between the two methods were found in most of the patients; in particular, FDOPA-PET/CT detected more lesions compared to FDG-PET/CT [16].

Marzola et al. [13] evaluated 18  patients who underwent both PET/CT methods for suspected MTC recurrence. These authors found a higher sensitivity of FDOPA-PET/CT compared to FDG PET/CT on a per-patient-based analysis (83% versus 61%, resp.). Discordant results were found in 6 cases (33%): in particular 5 patients were positive at FDOPA-PET/CT alone and one patient was positive at FDG-PET/CT alone [13].

Recently, Kauhanen et al. [6] evaluated 19  recurrent MTC patients with both methods, reporting a superiority of FDOPA-PET/CT compared to FDG-PET/CT (sensitivity on a per-patient-based analysis was 58% versus 53%, resp.). For most MTC patients with occult disease, FDOPA-PET/CT accurately detected metastases. In patients with an unstable calcitonin level, FDOPA-PET/CT and FDG-PET/CT were complementary. For patients with an unstable CEA doubling time, FDG-PET/CT was more feasible [6].

Lastly, in a recent multicentric study [5], 18 recurrent MTC performed both PET/CT methods. The sensitivity of FDOPA-PET/CT was superior compared to FDG-PET/CT on a per-patient-based analysis (72% versus 17%, resp.). Discordant results between FDOPA-PET/CT and FDG-PET/CT were found in 10/18 patients (56%), in whom FDOPA-PET/CT was positive and FDG-PET/CT was negative for MTC recurrence [5].

**(C) PET and PET/CT Using Other Radiopharmaceuticals**

Neuroendocrine tumors usually overexpress somatostatin receptors on their cell surface and this represents the rationale for using somatostatin analogues for diagnosis and therapy of these tumors. In fact, PET or PET/CT using somatostatin analogues labelled with Gallium-68 are valuable diagnostic tools for patients with neuroendocrine tumors [42]. Nevertheless, the experience with somatostatin analogues PET tracers in recurrent MTC is very limited [5, 9, 15]. A recent study comparing FDOPA, FDG, and somatostatin analogues labelled with Gallium-68 in recurrent MTC showed a significantly lower sensitivity of somatostatin receptor PET/CT (33%) compared to FDOPA-PET/CT (72%) [5]. Another study reported a complementary role of somatostatin receptor PET/CT compared to FDG-PET/CT in recurrent MTC [15].

However, somatostatin receptor PET could be a useful method in selecting patients for radioreceptor therapy to treat metastatic lesions showing a high expression of somatostatin receptors.

Lastly, Carbon-11-Methionine, a PET radiopharmaceutical used to evaluate the amino acid metabolism, was also used in detecting recurrent MTC, without significant advantages compared to FDG [10].

## 4. Conclusion and Future Perspectives

PET radiopharmaceuticals reflect different metabolic pathways and seem to show complementary role in detecting recurrent MTC.

There is an increasing evidence in the literature about the role of FDG-PET and PET/CT in recurrent MTC. FDG-PET and PET/CT should not be considered as first-line diagnostic imaging methods in patients with suspected recurrent MTC, but could be very helpful in detecting recurrence in those patients in whom a more aggressive disease is suspected.

To date, FDOPA seems to be the most useful PET radiopharmaceutical in detecting recurrent MTC based on rising levels of tumor markers. Nevertheless, the literature focusing on the use of FDOPA-PET or PET/CT in the detection of recurrent MTC remains still limited.

Other PET radiopharmaceuticals, such as somatostatin analogues labelled with Gallium-68, were also evaluated for this indication in a limited number of studies.

Multicenter and prospective studies investigating a larger patient population and comparing different PET radiopharmaceuticals in recurrent MTC are needed.

## Figures and Tables

**Table 1:** Basic study and patient characteristics.

Authors	Year	Country	MTC patients performing PET for suspected recurrence	Mean age (years)	% Male	Tracers used for PET or PET/CT
Treglia et al. [5]	2012	Italy	18	53	33%	FDG, FDOPA, and Gallium-68-DOTANOC/DOTATOC
Kauhanen et al. [6]	2011	Finland	19	52	53%	FDG and FDOPA
Ozkan et al. [7]	2011	Turkey	33	50	27%	FDG
Gómez-Camarero et al. [8]	2011	Spain	31	56	45%	FDG
Palyga et al. [9]	2010	Poland	8	56	50%	Gallium-68-DOTATATE
Jang et al. [10]	2010	Korea	16	51	56%	FDG and Carbon-11-methionine
Luster et al. [11]	2010	Germany	28	48	46%	FDOPA
Skoura et al. [12]	2010	Greece	32 (38 scans)	52	31%	FDG
Marzola et al. [13]	2010	Italy	18	51	44%	FDG and FDOPA
Bogsrud et al. [14]	2010	USA and Norway	29	50	55%	FDG
Conry et al. [15]	2010	UK and Singapore	18	54	72%	FDG and Gallium-68-DOTATATE
Beheshti et al. [16]	2009	Austria	19^∗^	59	38%	FDG and FDOPA
Faggiano et al. [17]	2009	Italy	26	NR	49%	FDG
Koopmans et al. [18]	2008	The Netherlands	21	56	48%	FDG and FDOPA
Rubello et al. [19]	2008	Italy	19	53	42%	FDG
Oudoux et al. [20]	2007	France	33	53	64%	FDG
Giraudet et al. [21]	2007	France	55	56	62%	FDG
Czepczyński et al. [22]	2007	Poland and Italy	13^∗^	50	57%	FDG
Beuthien-Baumann et al. [23]	2007	Germany	15	56	53%	FDG and FDOPA
Ong et al. [24]	2007	USA	28 (38 scans)	59	64%	FDG
Iagaru et al. [25]	2007	USA	13	48	46%	FDG
Gotthardt et al. [26]	2006	Germany and the Netherlands	26	45	58%	FDG
De Groot et al. [27]	2004	The Netherlands	26	51	58%	FDG
Szakáll et al. [28]	2002	Hungary	40	48	45%	FDG
Diehl et al. [29]	2001	Germany	85 (100 scans)	53	47%	FDG
Hoegerle et al. [30]	2001	Austria	10^∗^	57	55%	FDG and FDOPA
Brandt-Mainz et al. [31]	2000	Germany	17	NR	65%	FDG
Adams et al. [32]	1998	Germany	8	49	50%	FDG
Musholt et al. [33]	1997	USA and Germany	10	36	70%	FDG

NR: not reported; FDG: fluorine-18-fluorodeoxyglucose; FDOPA: fluorine-18-dihydroxyphenylalanine; ^∗^patients evaluated before primary surgery were excluded from the analysis.

**Table 2:** Technical aspects of the studies which used FDG-PET or PET/CT for detecting recurrent medullary thyroid carcinoma.

Authors	Study design	Device	Injected activity	Time between tracer injection and image acquisition (min)	PET acquisition protocol	Image analysis	Reference standard	Sensitivity of FDG-PET or PET/CT^∗^	Specificity of FDG-PET or PET/CT^∗^
Treglia et al. [5]	Retrospective multicenter	PET/CT	259–407 MBq	60	Static acquisition	Qualitative	Histology and/or clinical/imaging followup	17%	NC
Kauhanen et al. [6]	Prospective multicenter	PET/CT	377 MBq	60	Static acquisition (3 min per bed position)	Qualitative and semiquantitative	Histology and/or clinical/imaging followup	53%	NC
Ozkan et al. [7]	Retrospective single center	PET/CT	296–370 MBq	60	Static acquisition (4 min per bed position)	Qualitative and semiquantitative	Histology and/or clinical/imaging followup	93%	68%
Gómez-Camarero et al. [8]	Retrospective single center	PET and PET/CT	333–434 MBq	60	Static acquisition	Qualitative and semiquantitative	Histology and/or clinical/imaging followup	88%	85%
Jang et al. [10]	Prospective single center	PET/CT	370 MBq	60	Static acquisition (4 min per bed position)	Qualitative	Histology and/or clinical/imaging followup	63%	NC
Skoura et al. [12]	Retrospective single center	PET/CT	370 MBq	60	Static acquisition (4 min per bed position)	Qualitative and semiquantitative	Histology and/or clinical/imaging followup	47%	NC
Marzola et al. [13]	NR; multicenter	PET/CT	2.2 MBq/kg	60	Static acquisition (3 min per bed position)	Qualitative and semiquantitative	Histology	61%	NC
Bogsrud et al. [14]	Retrospective single center	PET and PET/CT	740 MBq	60–75	Static acquisition (5 min per bed position)	Qualitative	Histology and/or clinical/imaging followup	45%	93%
Conry et al. [15]	Retrospective multicenter	PET/CT	195–550 MBq	50–75	Static acquisition (1.5/5 min per bed position)	Qualitative and semiquantitative	Histology and/or clinical/imaging followup	78%	NC
Beheshti et al. [16]	Prospective single center	PET/CT	370 MBq	60	Static acquisition (4 min per bed position)	Qualitative and semiquantitative	Histology and/or clinical/imaging followup	58%	NC
Faggiano et al. [17]	Retrospective multicenter	PET	222–370 MBq	60–90	Static acquisition (4 min per bed position)	Qualitative	Histology and/or clinical/imaging followup	50%	NC
Koopmans et al. [18]	Prospective single center	PET	NR	NR	Static acquisition(5 min per bed position)	Qualitative	Histology and/or clinical/imaging followup	24%	NC
Rubello et al. [19]	Prospective multicenter	PET/CT	5.5 MBq/kg	60–90	Static acquisition (4 min per bed position)	Qualitative and semiquantitative	Histology	79%	100%
Oudoux et al. [20]	Prospective multicenter	PET/CT	310–450 MBq	60	Static acquisition	Qualitative and semiquantitative	Histology and/or clinical/imaging followup	76%	NC
Giraudet et al. [21]	Prospective single center	PET/CT	5 MBq/Kg	60	Static acquisition	Qualitative and semiquantitative	Histology and/or clinical/imaging followup	32%	NC
Czepczyński et al. [22]	NR; single center	PET	NR	NR	Static acquisition	Qualitative	Histology and/or clinical/imaging followup	58%	NC
Beuthien-Baumann et al. [23]	Retrospective single center	PET	370 MBq	60	Static acquisition	Qualitative	Histology and/or clinical/imaging followup	47%	NC
Ong et al. [24]	Retrospective single center	PET and PET/CT	555 MBq	Minimum 45	Static acquisition (4 min per bed position)	Qualitative and semiquantitative	Histology and/or clinical/imaging followup	62%	NC
Iagaru et al. [25]	Retrospective single center	PET and PET/CT	550 MBq	45/60	Static acquisition (4/5 min per bed position)	Qualitative	Histology and/or clinical/imaging followup	86%	83%
Gotthardt et al. [26]	NR; multicenter	PET	350 MBq	60	Static acquisition	Qualitative	Histology and/or clinical/imaging followup	70%	NC
De Groot et al. [27]	Prospective single center	PET	400 MBq	90	Static acquisition (5 min per bed position)	Qualitative	Histology and/or clinical/imaging followup	41%	NC
Szakáll et al. [28]	Retrospective single center	PET	5.55 MBq/Kg	40	Static acquisition (10 min per bed position)	Qualitative	Histology and/or clinical/imaging followup	95%	NC
Diehl et al. [29]	Retrospective multicenter	PET	300–500 MBq	Minimum 30	Static acquisition	Qualitative	Histology and/or clinical/imaging followup	78%	79%
Hoegerle et al. [30]	Prospective single center	PET	330 MBq	90	Static acquisition	Qualitative	Histology and/or clinical/imaging followup	60%	100%
Brandt-Mainz et al. [31]	Prospective single center	PET	350 MBq	30	Static acquisition (15–20 min per bed position)	Qualitative	Histology and/or clinical/imaging followup	76%	NC
Adams et al. [32]	Prospective single center	PET	374 MBq	60	Static acquisition (12–15 min per bed position)	Qualitative	Histology and/or clinical/imaging followup	87%	NC
Musholt et al. [33]	NR; single center	PET	370–555 MBq	40	Static acquisition (10 min per bed position)	Qualitative	Histology and/or clinical/imaging followup	90%	NC

NR: not reported; NC: not calculated; ^∗^sensitivity and specificity are reported on a per patient-based analysis.

**Table 3:** Technical aspects of the studies which used FDOPA-PET or PET/CT for detecting recurrent medullary thyroid carcinoma.

Authors	Study design	Device	Injected activity	Time between tracer injection and image acquisition (min)	PET acquisition protocol	Image analysis	Reference standard	Sensitivity of FDOPA-PET or PET/CT^∗^	Specificity of FDOPA-PET or PET/CT^∗^
Treglia et al. [5]	Retrospective multicenter	PET/CT	4 MBq/kg	60	Static acquisition (3 min per bed position)no carbidopa premedication	Qualitative	Histology and/or clinical/imaging followup	72%	NC
Kauhanen et al. [6]	Prospective multicenter	PET/CT	243 MBq	60	Static acquisition (3 min per bed position)carbidopa premedication	Qualitative and semiquantitative	Histology and/or clinical/imaging followup	58%	NC
Luster et al. [11]	Retrospective single center	PET/CT	298 MBq	60	Static acquisition (4 min per bed position)carbidopa premedication	Qualitative and semiquantitative	Histology and/or clinical/imaging followup	74%	100%
Marzola et al. [13]	Multicenter	PET/CT	2.2 MBq/kg	60	Static acquisition (3 min per bed position)no carbidopa premedication	Qualitative and semiquantitative	Histology	83%	NC
Beheshti et al. [16]	Prospective single center	PET/CT	4 MBq/Kg	30	Static acquisition (4 min per bed position)no carbidopa premedication	Qualitative and semiquantitative	Histology and/or clinical/imaging followup	81%	NC
Koopmans et al. [18]	Prospective single center	PET	180 MBq	60	Static acquisition; (5 min per bed position)carbidopa premedication	Qualitative	Histology and/or clinical/imaging followup	62%	NC
Beuthien-Baumann et al. [23]	Retrospective single center	PET	4.8 MBq/Kg	45	Static acquisition carbidopa premedication	Qualitative	Histology and/or clinical/imaging followup	47%	NC
Hoegerle et al. [30]	Prospective single center	PET	220 MBq	90	Static acquisition no carbidopa premedication	Qualitative	Histology and/or clinical/imaging followup	60%	NC

NC: not calculated; ^∗^sensitivity and specificity are reported on a per patient-based analysis.
